# Melatonin Alleviates Photosynthetic Injury in Tomato Seedlings Subjected to Salt Stress via OJIP Chlorophyll Fluorescence Kinetics

**DOI:** 10.3390/plants14050824

**Published:** 2025-03-06

**Authors:** Xianjun Chen, Xiaofeng Liu, Yundan Cong, Yao Jiang, Jianwei Zhang, Qin Yang, Huiying Liu

**Affiliations:** 1 Provincial Famous Teacher Yang Qin Studio/Key Laboratory of Molecular Breeding and Variety Creation of Horticultural Plants for Mountain Features in Guizhou Province, School of Life and Health Science, Kaili University, Kaili 556011, China; chenxianjun_0805@163.com (X.C.); lxf202407@163.com (X.L.); jiangyao0221@163.com (Y.J.); zhangjw1831@163.com (J.Z.); 2 Key Laboratory of Special Fruits and Vegetables Cultivation Physiology and Germplasm Resources Utilization of Xinjiang Production and Contruction Crops, Department of Horticulture, Agricultural College, Shihezi University, Shihezi 832003, China; yundan_cong@163.com

**Keywords:** melatonin (MT), OJIP, salt stress, tomato, photosynthetic

## Abstract

The tomato is among the crops with the most extensive cultivated area and greatest consumption in our nation; nonetheless, secondary salinization of facility soil significantly hinders the sustainable growth of facility agriculture. Melatonin (MT), as an innovative plant growth regulator, is essential in stress responses. This research used a hydroponic setup to replicate saline stress conditions. Different endogenous levels of melatonin (MT) were established by foliar spraying of 100 μmol·L^−1^ MT, the MT synthesis inhibitor p-CPA (100 μmol·L^−1^), and a combination of p-CPA and MT, to investigate the mechanism by which MT mitigates the effects of salt stress on the photosynthetic efficiency of tomato seedlings. Results indicated that after six days of salt stress, the endogenous MT content in tomato seedlings drastically decreased, with declines in the net photosynthetic rate and photosystem performance indices (PI_total_ and PI_abs_). The OJIP fluorescence curve exhibited distortion, characterized by anomalous K-band and L-band manifestations. Exogenous MT dramatically enhanced the gene (*TrpDC*, *T5H*, *SNAcT*, and *AcSNMT*) expression of critical enzymes in MT synthesis, therefore boosting the level of endogenous MT. The application of MT enhanced the photosynthetic parameters. MT treatment decreased the fluorescence intensities of the J-phase and I-phase in the OJIP curve under salt stress, attenuated the irregularities in the K-band and L-band performance, and concurrently enhanced quantum yield and energy partitioning ratios. It specifically elevated φP_o_, φE_o_, and ψ_o,_ while decreasing φD_o_. The therapy enhanced parameters of both the membrane model (ABS/RC, DI_o_/RC, ET_o_/RC, and TR_o_/RC) and leaf model (ABS/CS_m_, TR_o_/CS_m_, ET_o_/CS_m_, and DI_o_/CS_m_). Conversely, the injection of exogenous p-CPA exacerbated salt stress-related damage to the photosystem of tomato seedlings and diminished the beneficial effects of MT. The findings suggest that exogenous MT mitigates salt stress-induced photoinhibition by (1) modulating endogenous MT concentrations, (2) augmenting PSII reaction center functionality, (3) safeguarding the oxygen-evolving complex (OEC), (4) reinstating PSI redox potential, (5) facilitating photosynthetic electron transport, and (6) optimizing energy absorption and dissipation. As a result, MT markedly enhanced photochemical performance and facilitated development and salt stress resilience in tomato seedlings.

## 1. Introduction

China is the world’s greatest producer of vegetables, with facility agriculture accounting for almost one-third of output [1,2]. In recent years, protected cultivation has grown significantly, becoming a modernized and effective farming method that significantly supports sustainable agricultural development [3,4]. Facility vegetables play a vital role in altering rural industrial systems and raising farmers’ incomes, yet they also confront several obstacles. Among them, the problem of secondary salinization induced by inappropriate irrigation and fertilization techniques has grown more serious, acting as a significant hurdle to the growth of protected agriculture [5,6,7,8]. Plants under salt stress experience osmotic stress and ion toxicity, which have an indirect impact on energy metabolism, photosynthesis, membrane permeability, and overall growth and development [9,10,11]. Soil salinization has emerged as a primary bottleneck restricting the sustainable development of facility and modern agriculture. Photosynthesis supplies the essential energy and materials for plant development, but salt stress may significantly hinder photosynthetic capability by interrupting the photosynthetic electron transport chain, leading to photoinhibition [12,13,14]. This eventually inhibits plant development and adversely affects agricultural output and quality. The tomato (*Solanum lycopersicum*), a salt-sensitive plant, is a crucial crop in controlled environment agriculture, with its productivity and quality markedly impacted by salt stress [15]. A thorough investigation of the physiological response mechanisms of tomatoes to salt stress and the regulatory pathways of salt tolerance is essential for breeding salt-tolerant varieties and offers a scientific foundation for formulating effective cultivation management strategies, thus advancing the efficient and sustainable development of facility agriculture.

Melatonin (MT) is an indoleamine that is prevalent in both mammals and plants. It has been identified as a possible plant hormone that modulates responses to diverse biotic and abiotic stresses [16,17,18]. Since both MT and auxin (IAA) are derived from the same precursor, tryptophan, they exhibit similar functional roles in plants [19,20,21]. As a growth regulator, MT enhances plant development by modulating growth processes [22]. Additionally, MT serves as a powerful antioxidant, playing a vital role in the organism’s defense system against oxidative stress. It achieves this by scavenging excess reactive oxygen species (ROS), stimulating the activity of antioxidant enzymes, suppressing pro-oxidant enzymes, and minimizing lipid peroxidation [23,24,25]. Through its regulation of hormone signaling pathways, transcription of pertinent genes, and the production and degradation of chlorophyll, MT dramatically increases the photosynthetic capability of plants under abiotic stress [26]. By activating antioxidant systems, promoting the transcription of genes linked to photosynthesis, and promoting the xanthophyll cycle, it safeguards the function of photosynthetic enzymes and ensures effective photosynthesis [27,28]. Additionally, via influencing sugar, gluconeogenesis, and the breakdown and transport of transitory starch, MT controls the photosynthetic carbon cycle and affects stomatal movement by modifying the CAND2/PMTR1 pathway [27]. According to research, exogenous melatonin increases the photochemical activity of both photosystem I (PSI) and photosystem II (PSII), which boosts cold tolerance and enhances the photosynthetic efficiency of pepper (*Capsicum annuum*) seedlings [29]. The photochemical activity of the PSII reaction center is maintained in wheat (*Triticum aestivum*) when MT promotes the dephosphorylation of thylakoid membrane proteins (LHCII, CP43, and D1) and upregulates the transcription of PSII-related proteins (D1, Lhcb5, Lhcb6, PsbQ, and PsbS) under osmotic stress [30]. Furthermore, by increasing the activity of antioxidant enzymes, exogenous MT maintains the photochemical activity of PSII in maize (*Zea mays*) seedlings under NaCl stress [31].

The chlorophyll a fluorescence OJIP transient approach is a quick, easy, and non-invasive way to determine the oxidation-reduction status of PSI and PSII photosystems. It has been used to track physiological changes in plants under stress and assess the degree of damage to the photosynthetic apparatus [32,33,34]. Although previous studies have shown that melatonin (MT) can enhance salt tolerance by boosting antioxidant capacity, regulating ion and osmotic balance, and interacting with other hormones—thereby improving PSII photochemical efficiency under salt stress [35,36,37,38]—most investigations have focused on overall photosynthetic performance rather than a detailed analysis of the redox states and energy distribution within the photosystems. As a result, this experiment used hydroponic culture with simulated salt conditions and exogenously sprayed MT, p-CPA (MT synthesis inhibitor), and MT+p-CPA to establish tomato plants that had differing endogenous MT levels. The effects of MT on the oxidation-reduction state of photosystems under salt stress, the photosynthetic electron transport chain, and the stability of the photosynthetic system were examined using OJIP transient analysis. The research indicates that exogenous MT improves the stability of PSII and PSI by augmenting endogenous MT, modulates photosynthetic activity and energy allocation efficiency, and may substantially mitigate the adverse effects of salt stress on photosynthetic processes and plant development. This study not only advances our understanding of the specific mechanisms by which MT modulates photosystem stability but also provides new insights into its potential application for improving salt tolerance in tomato seedlings.

## 2. Materials and Methods

### 2.1. Plant Materials and Treatment Conditions

This experiment used the hydroponically grown tomato seedlings (*Solanum lycopersicum* L. cv. ‘Zhongshu No. 4’), which were grown in a greenhouse at Kaili University in Guizhou, China. Germinated tomato seeds with radicles were placed in seedling trays with peat and vermiculite (2:1, *v*/*v*) for early development. Uniform two-leaf, one-heart seedlings were placed in 12 L black plastic containers with foam lids and 10 L of Hoagland nutrition solution made with deionized water (pH = 6.2). Treatments followed a 7-day pre-culture. Melatonin (MT) and p-CPA (4-chloro-DL-phenylalanine, a MT synthesis inhibitor) were sprayed on the leaves after adding NaCl to the nutrition solution. The five treatments that were used in this study were as follows: (1) the control, which consisted of the absence of any additional NaCl, p-CPA, or MT; (2) 100 mmol·L^−1^ NaCl (NaCl); (3) 100 mmol·L^−1^ NaCl with 100 μmol·L^−1^ MT (NM); (4) 100 mmol·L^−1^ NaCl together with 100 μmol·L^−1^ p-CPA (NP); and (5) 100 mmol·L^−1^ NaCl, 100 μmol·L^−1^ MT, and 100 μmol·L^−1^ p-CPA (NMP). After NaCl treatment, we sprayed 100 µmol·L^−1^ MT immediately (Day 0), followed by five additional sprays at 24-h intervals, resulting in a total of six applications. MT and p-CPA were acquired from Sigma (St. Louis, MI, USA) and Macklin (Shanghai, China), respectively. MT and p-CPA concentrations and volumes were based on an unpublished research. With five plants per container and four replicates per treatment, a randomized full block design was used. The seedlings were subjected to 14-h light cycles every day, with daytime temperatures of 24–30 °C and nighttime temperatures of 17–20 °C. On the sixth day of treatment, leaf samples were taken, and nutrient solutions were changed every two days.

### 2.2. Measurement of Growth

Plant height was measured from the cotyledonary node to the growth point, while stem diameter was recorded at the cotyledonary node. The shoot and root portions were separated to determine fresh weights. The samples were dried at 105 °C for 15 min, then at 75 °C to a constant weight, and weighed.

### 2.3. MT Content

Following the protocol described by Yang et al. [39], 0.1 g of leaf tissue was combined with 1 mL of 80% methanol, thoroughly homogenized, and incubated overnight to facilitate extraction. The sample was then centrifuged, and the resulting supernatant was passed through a needle filter. Endogenous MT content in the filtrate was quantified using a RIGOL L3000 high-performance liquid chromatograph (RIGOL Technologies, Beijing, China) coupled with a Shimadzu RF-20A fluorescence detector (Shimadzu Corporation, Kyoto, Japan), with excitation at 280 nm and emission at 348 nm. Chromatographic separation was performed on a RIGOL C18 reversed-phase column (250 mm × 4.6 mm, 5 µm) at 30 °C, with a flow rate of 0.8 mL·min^−1^ and an injection volume of 10 µL. The mobile phase consisted of solvent A (methanol) and solvent B (0.1% formic acid) under the following gradient program: 0–10 min: A maintained at 10%; 10–30 min: A increased from 10% to 50%; 30–35 min: A decreased from 50% back to 10%; 35–45 min: A maintained at 10%.

### 2.4. Measurement of Photosynthetic Parameters

On the sixth day after treatment, the fourth functional leaf (from the top) of the tomato seedlings was chosen to measure photosynthetic gas-exchange parameters. The measurements were conducted between 8:00 and 10:00 AM using a Li-6400XT portable photosynthesis system (Li-COR, Lincoln, NE, USA). The following characteristics were measured: net photosynthetic rate (*P*_n_), stomatal conductance (*G*_s_), intercellular CO_2_ concentration (*C*_i_), and transpiration rate (*T*_r_). Five measurements were made for each replication of each treatment, which was carried out in triplicate. The experimental parameters were maintained at 25 °C, 1000 ± 10 µmol·m^2^·s^−1^ of light intensity, and 350 ± 5 µmol·mol^−1^ of atmospheric CO_2_.

### 2.5. OJIP Transient Measurement and Analysis in Fast Fluorescence Induction Kinetics

OJIP curves for the fourth functional leaf were assessed using the M-PEA Multifunctional Plant Efficiency Analyzer (Hansatech, Birmingham, UK) under saturating pulsed light (5000 µmol·m^2^·s^−1^) after a two-hour dark adaption. At an initial recording rate of 1 × 10^5^ data points per second, fluorescence signals were recorded for up to 3 s. According to Lima-Moro’s methodology [40], the JIP-test analysis was conducted. Supplementary Table S1 lists the JIP-test parameters together with the formulae used to calculate them.

### 2.6. Quantitative Polymerase Chain Reaction (qPCR) Methodology

The tomato seedlings’ gene expression levels were assessed using quantitative polymerase chain reaction, or qPCR. The fourth functioning leaf was used to extract total RNA using the Trizol technique (Thermo-Fisher Scientific, San Jose, CA, USA). The Hyper Script™ III RT SuperMix (EnzyArtisan Biotech, Shanghai, China) was used to synthesize cDNA from RNA, following the manufacturer’s instructions. A 96-well plate was used to create the qPCR reaction, which contained a no-template control, gene-specific primers, cDNA, nuclease-free water, and 2 × S6 Universal SYBR qPCR Mix (Enzyme Biotech, Beijiing, China). After a 10-min denaturation at 95 °C, 40 cycles of 15-s denaturation, 30-s annealing at a particular temperature, and 30-s extension at 72 °C were performed. To confirm the amplified products’ specificity, a melt curve analysis was performed. The tomato actin gene was used as an internal control in the 2^−ΔΔCt^ technique to determine relative gene expression levels [41]. Three biological duplicates were utilized in the experiment, and Supplementary Table S2 lists the precise primer sequences that were employed to amplify the target genes.

### 2.7. Statistical Analysis

SPSS 19 (IBM Corporation, Chicago, IL, USA) and Microsoft Office Excel 2020 (Microsoft Corporation, Washington, DC, USA) were used for data analysis, and Origin 2018 (OriginLab, Hampton, MA, USA) was used to create graphics. Prior to conducting one-way analysis of variance (ANOVA), the Shapiro–Wilk test was applied to assess normality, and Levene’s test was used to verify homogeneity of variances. The mean values of each treatment were then assessed using ANOVA, followed by pairwise comparisons using Tukey’s HSD test to minimize the risk of Type I errors. Statistical significance was set at *p* < 0.05.

## 3. Results

### 3.1. Effects of Exogenous MT on the Growth of Tomato Seedlings Under Salt Stress

**Figure 1:**
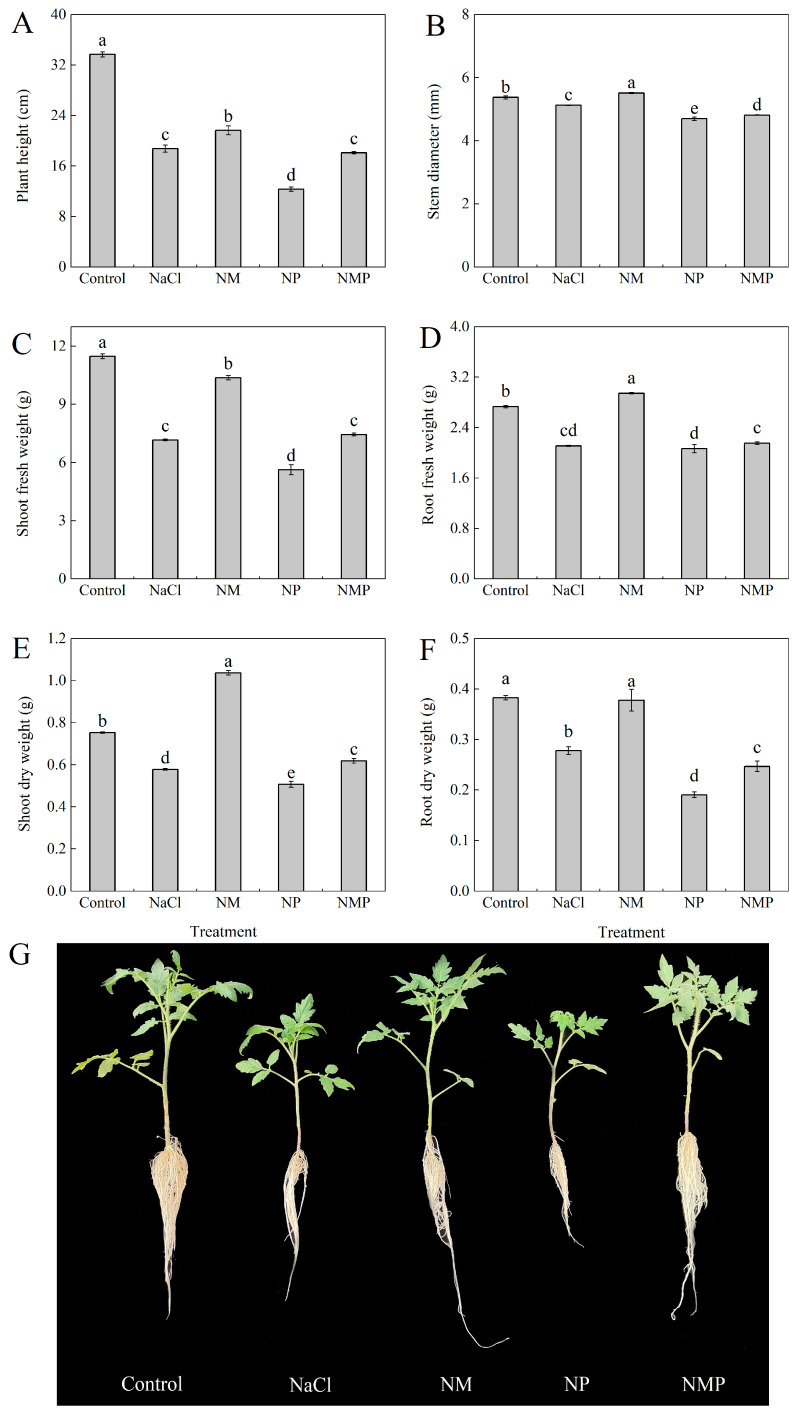
Effects of exogenous melatonin (MT) on tomato seedlings under salt stress: plant height (**A**), stem diameter (**B**), shoot fresh weight (**C**), root fresh weight (**D**), shoot dry weight (**E**), root dry weight (**F**), and photographs of tomato seedlings under different treatments (**G**). Treatment differences are significant when indicated by different lowercase letters (*p* < 0.05). Control: No NaCl, p-CPA, or MT added. NaCl: 100 mmol·L^−1^ NaCl applied alone. NM: 100 mmol·L^−1^ NaCl + 100 μmol·L^−1^ MT applied. NP: 100 mmol·L^−1^ NaCl + 100 μmol·L^−1^ p-CPA (MT synthesis inhibitor) applied. NMP: 100 mmol·L^−1^ NaCl + 100 μmol·L^−1^ MT + 100 μmol·L^−1^ p-CPA applied.

Salt stress induced by NaCl significantly inhibited the growth of the tomato seedlings, as evidenced by reductions in plant height, stem diameter, shoot fresh weight, root fresh weight, shoot dry weight, and root dry weight (Figure 1). Compared to the control group, these parameters decreased by 44.3%, 4.5%, 37.6%, 22.8%, 23.3%, and 27.4%, respectively. However, the application of exogenous melatonin (MT) markedly alleviated these adverse effects, resulting in increases of 15.4% in plant height, 7.5% in stem diameter, 44.9% in shoot fresh weight, 39.5% in root fresh weight, 79.6% in shoot dry weight, and 35.9% in root dry weight, respectively. In contrast, the addition of p-CPA, an MT synthesis inhibitor, exacerbated the inhibitory effects of salt stress on seedling growth, further reducing the measured parameters. Moreover, when p-CPA was applied following the MT treatment, the mitigating effects of MT on seedling growth were significantly diminished.

### 3.2. Effects of Exogenous MT on the Endogenous MT Content of Tomato Seedlings Under Salt Stress

**Figure 2:**
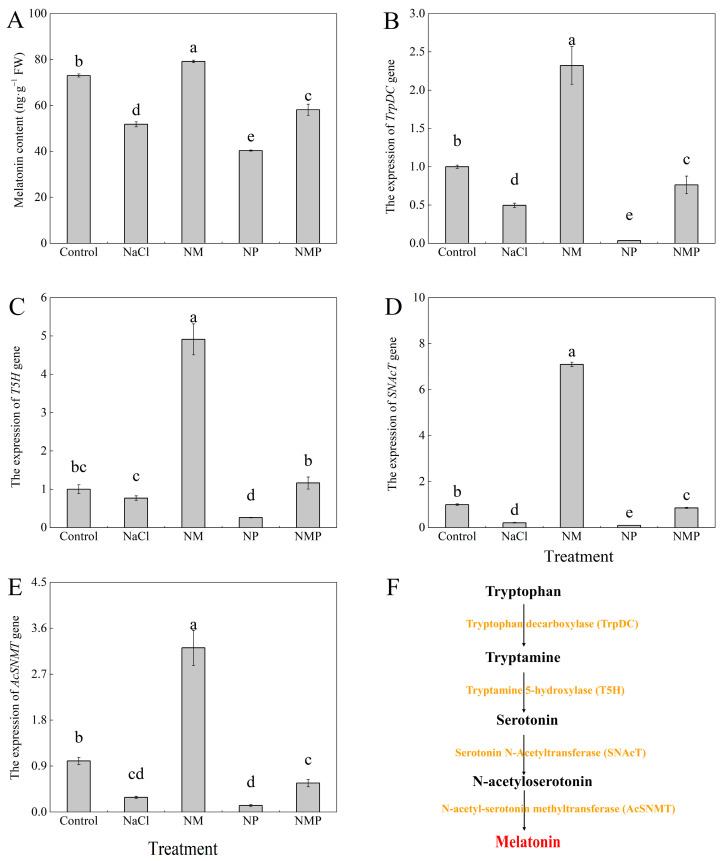
Effects of exogenous melatonin (MT) on tomato seedlings under salt stress: melatonin content (**A**), the expression of *TrpDC* gene (**B**), the expression of *TH5* gene (**C**), the expression of SNAcT gene (**D**), and the expression of AcSNMT gene (**E**), accompanied by a schematic illustration of the melatonin biosynthesis pathway (**F**). Treatment differences are significant when indicated by different lowercase letters (*p* < 0.05). Control: No NaCl, p-CPA, or MT added. NaCl: 100 mmol·L^−1^ NaCl applied alone. NM: 100 mmol·L^−1^ NaCl + 100 μmol·L^−1^ MT applied. NP: 100 mmol·L^−1^ NaCl + 100 μmol·L^−1^ p-CPA (MT synthesis inhibitor) applied. NMP: 100 mmol·L^−1^ NaCl + 100 μmol·L^−1^ MT + 100 μmol·L^−1^ p-CPA applied.

The endogenous MT content in tomato seedlings was significantly decreased by salt stress, as shown in Figure 2, which also showed a significant downregulation of the expression of genes associated with MT synthesis (*TrpDC*, *T5H*, *SNAcT*, *AcSNMT*). Following the administration of exogenous MT, the MT content exhibited a considerable increase, demonstrating a 52.9% elevation relative to the NaCl treatment alone. Moreover, gene expression levels were significantly elevated, with TrpDC expression increasing by 3.68-fold, T5H by 5.38-fold, SNAcT by 32.87-fold, and AcSNMT by 10.21-fold. Conversely, the application of p-CPA (an MT synthesis inhibitor) with NaCl resulted in considerably reduced endogenous MT content and decreased expression levels of MT synthesis-related genes compared to both the NaCl and NM (NaCl + MT) treatments.

### 3.3. Effects of Exogenous MT on the Photosynthetic Parameter of Tomato Seedlings Under Salt Stress

**Figure 3:**
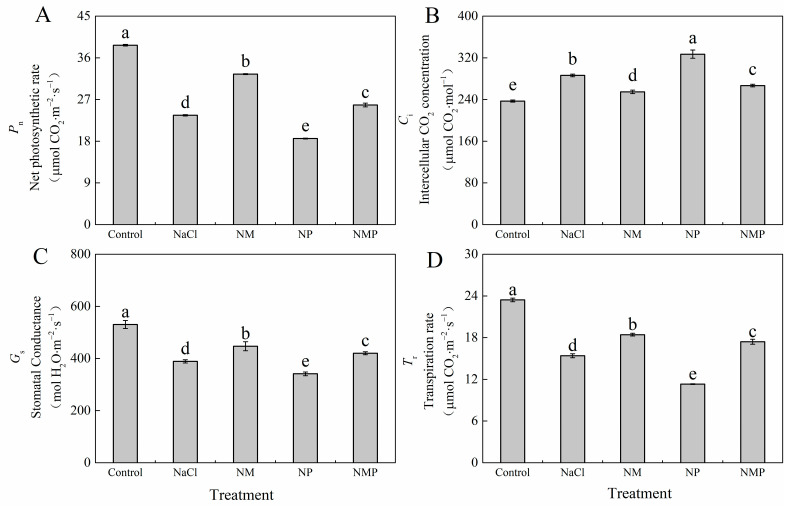
Effects of exogenous melatonin (MT) on tomato seedlings under salt stress: the net photosynthetic rate (*P*_n_) (**A**), the intercellular CO_2_ concentration (*C*_i_) (**B**), stomatal conductance (*G*_s_) (**C**), and transpiration rate (*T*_r_) (**D**). Treatment differences are significant when indicated by different lowercase letters (*p* < 0.05). Control: No NaCl, p-CPA, or MT added. NaCl: 100 mmol·L^−1^ NaCl applied alone. NM: 100 mmol·L^−1^ NaCl + 100 μmol·L^−1^ MT applied. NP: 100 mmol·L^−1^ NaCl + 100 μmol·L^−1^ p-CPA (MT synthesis inhibitor) applied. NMP: 100 mmol·L^−1^ NaCl + 100 μmol·L^−1^ MT + 100 μmol·L^−1^ p-CPA applied.

In tomato seedling leaves, Figure 3 demonstrates that by day six, the NaCl treatment dramatically increased the *C*_i_ while drastically decreasing the *P*_n_, *G*_s_, and *T*_r_ in comparison to the control. Exogenous MT substantially reduced the NaCl’s deleterious effects on *P*_n_, *G*_s_, *T*_r_, and *C*_i_ (*p* < 0.05). Conversely, the NP treatment resulted in an additional reduction in *P*_n_, *G*_s_, and *T*_r_ compared to both the NaCl and NM treatments. The NP treatment reduced *P*_n_, *G*_s_, and *T*_r_ by 21.2%, 12.3%, and 26.5%, respectively, in comparison to the NaCl treatment alone, although *C*_i_ increased by 14.2%. During the NMP therapy, *P*_n_, *G*_s_, and *T*_r_ were reduced by 20.5%, 6.0%, and 5.6%, respectively, compared to the NM treatment, whereas *C*_i_ exhibited a 4.7% rise.

### 3.4. Effects of Exogenous MT on the OJIP Curves of Tomato Seedlings Under Salt Stress

**Figure 4:**
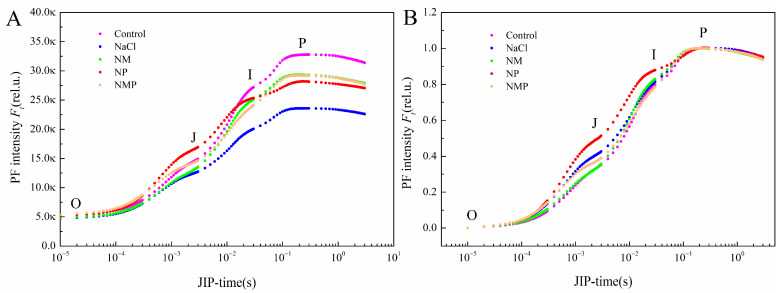
Effects of exogenous melatonin (MT) on tomato seedlings under salt stress: the rapid chlorophyll a fluorescence induction (OJIP) curves (**A**), and normalized transients between the O and P steps, expressed as V_t_ = [(F_t_ − F_o_)/(F_M_ − F_o_)] (**B**). Control: No NaCl, p-CPA, or MT added. NaCl: 100 mmol·L^−1^ NaCl applied alone. NM: 100 mmol·L^−1^ NaCl + 100 μmol·L^−1^ MT applied. NP: 100 mmol·L^−1^ NaCl + 100 μmol·L^−1^ p-CPA (MT synthesis inhibitor) applied. NMP: 100 mmol·L^−1^ NaCl + 100 μmol·L^−1^ MT + 100 μmol·L^−1^ p-CPA applied.

The OJIP transient fluorescence curves between the O and P phases under different treatments are presented in Figure 4A. The NaCl treatment reduced the PF intensity at the I and P phases and decreased the I-P amplitude. However, the application of MT effectively counteracted these NaCl-induced changes, resulting in higher PF intensities at the I and P phases and an increased I-P amplitude. In contrast, the NMP treatment led to higher PF intensity at the J phase but lower intensity at the I and P phases compared to the NM. Meanwhile, NP-treated seedlings exhibited significantly higher PF intensity between the O and P phases than those subjected to the NaCl treatment.

To further analyze the OJIP transient, the fluorescence measurements were normalized to a range of 0–1 (Figure 4B), enabling a detailed examination of the O-J, J-I, and I-P phases. Standardizing the O-P amplitude (WO-P) provided insights into changes in the electron transport chain on the acceptor side of PSII. As shown in Figure 4B, the NaCl treatment increased the PF intensity during the O-J phase compared to the control. In contrast, the exogenous MT application reduced the O-J phase intensity under salt stress. The addition of p-CPA further elevated the PF intensity during the O-J and J-I phases compared to the NaCl treatment. Additionally, the NMP treatment increased the PF intensity at the O-J phase relative to the NM.

**Figure 5:**
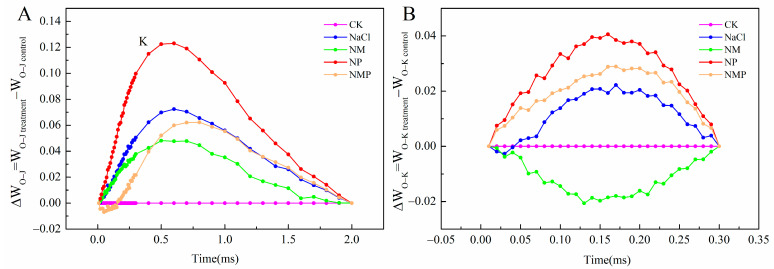
Effects of exogenous melatonin (MT) on tomato seedlings under salt stress: the differential kinetics of ∆W_O-J_ = W_O-J(stress)_ − W_O-J(control)_ over a linear time scale from 0 to 2 ms (**A**), and ∆W_O-K_ = W_O-K(stress)_ − W_O-K(control)_ over a linear time scale from 0 to 300 μs (**B**). Control: No NaCl, p-CPA, or MT added. NaCl: 100 mmol·L^−1^ NaCl applied alone. NM: 100 mmol·L^−1^ NaCl + 100 μmol·L^−1^ MT applied. NP: 100 mmol·L^−1^ NaCl + 100 μmol·L^−1^ p-CPA (MT synthesis inhibitor) applied. NMP: 100 mmol·L^−1^ NaCl + 100 μmol·L^−1^ MT + 100 μmol·L^−1^ p-CPA applied.

The K-band, which is the OJIP curve at 300 μs, is a particular measure of photoinhibition on the receptor side of PSII. As the K-band rises, it indicates the inactivation or degradation of the oxygen-evolving complex (OEC) [42]. The O-J phase of the OJIP curve was normalised to see the K-band (Figure 5A). The tomato seedlings’ leaves under salt stress had a K-band that was noticeably higher than the control’s. Furthermore, during salt stress, the K-band was considerably raised by the application of the p-CPA. Exogenous MT alleviated the elevation in the K-band caused by NaCl. The K-band in the NMP therapy was much higher than in the NM treatment.

Thylakoid disintegration is indicated by an increase in the L-band, which is the OJIP curve between 100 and 200 μs. The OJIP curve’s O-K phase was normalised in order to analyse the L-band (Figure 5B). Comparing the NaCl treatment to the control, it was found that the L-band at 150 μs was higher. Applying the MT synthesis inhibitor p-CPA in the NP treatment caused the L-band to significantly rise, but the NM treatment caused the L-band to diminish in comparison to the NaCl treatment. Furthermore, compared to the NM treatment, the L-band in the NMP therapy was much larger.

### 3.5. Effects of Exogenous MT on the JIP-Text Parameters of Tomato Seedlings Under Salt Stress

**Figure 6:**
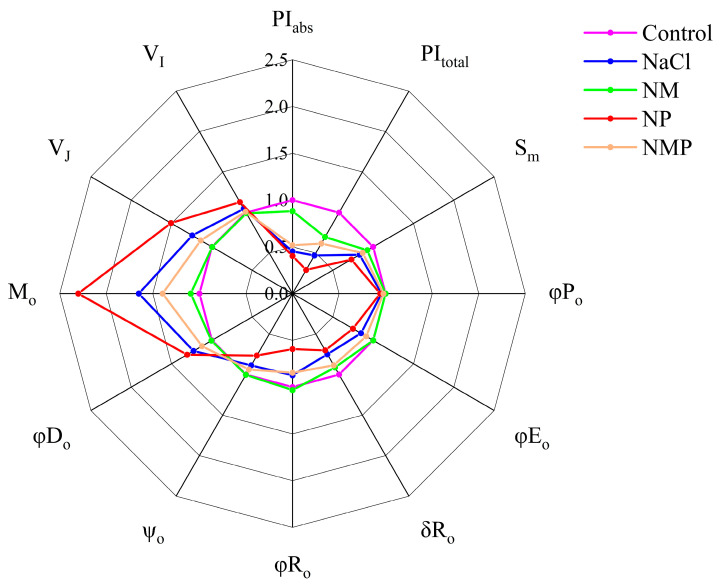
Effects of exogenous melatonin (MT) on JIP-test parameters derived from chlorophyll a fluorescence OJIP transients in tomato seedling leaves under salt stress, represented as spider plots. Control: No NaCl, p-CPA, or MT added. NaCl: 100 mmol·L^−1^ NaCl applied alone. NM: 100 mmol·L^−1^ NaCl + 100 μmol·L^−1^ MT applied. NP: 100 mmol·L^−1^ NaCl + 100 μmol·L^−1^ p-CPA (MT synthesis inhibitor) applied. NMP: 100 mmol·L^−1^ NaCl + 100 μmol·L^−1^ MT + 100 μmol·L^−1^ p-CPA applied.

A number of parameters derived from the OJIP curves show notable changes under various treatments, as seen in Figure 6. V_J_, V_I_, M_O,_ and φD_o_ were significantly higher after the NaCl treatment than after the Control, although PI_abs_, PI_total_, S_m_, φP_o_, φE_o_, δR_o_, φR_o_, and ψ_o_ were significantly lower. The exogenous MT application significantly counteracted these changes caused by NaCl, lowering V_J_, V_I_, M_O_, and φD_o_ by 20.2%, 5.4%, 34.5%, and 16.9%, respectively, while sharply raising PI_abs_, PI_total_, S_m_, φP_o_, φE_o_, δR_o_, φR_o_, and ψ_o_ by 98.3%, 49.4%, 11.5%, 4.2%, 17.3%, 20.8%, 18.6%, and 12.9%, respectively. Nevertheless, the administration of p-CPA reduced the mitigating effects of the MT (NMP treatment) and increased the damage caused by salt stress to tomato seedlings (NP treatment).

**Figure 7:**
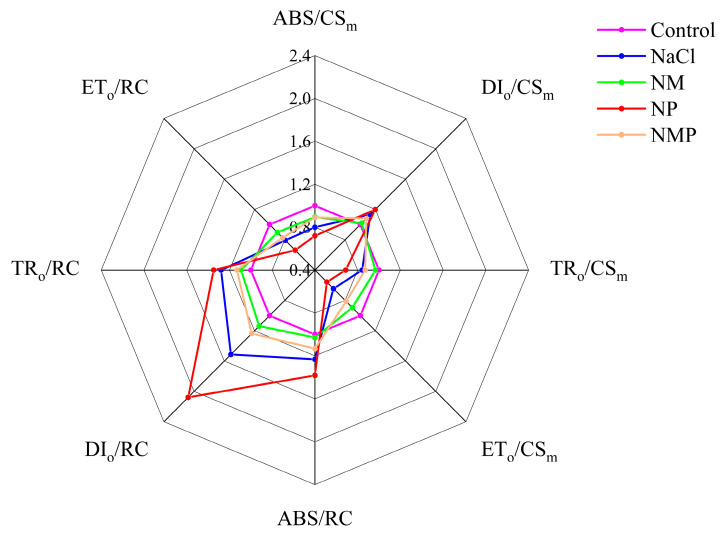
Effects of exogenous melatonin (MT) on energy distribution parameters per PSII reaction center (RC) and excitation cross-sectional area (CS_m_) in the leaves of tomato seedlings under salt stress, represented as spider plots. Control: No NaCl, p-CPA, or MT added. NaCl: 100 mmol·L^−1^ NaCl applied alone. NM: 100 mmol·L^−1^ NaCl + 100 μmol·L^−1^ MT applied. NP: 100 mmol·L^−1^ NaCl + 100 μmol·L^−1^ p-CPA (MT synthesis inhibitor) applied. NMP: 100 mmol·L^−1^ NaCl + 100 μmol·L^−1^ MT + 100 μmol·L^−1^ p-CPA applied.

In the tomato seedling leaves, ABS/RC, DI_o_/RC, and TR_o_/RC rose by 23.2%, 51.2%, and 27.9%, respectively, under 100 mM NaCl stress, but ET_o_/RC dramatically reduced by 20.8% (Figure 7). These effects were reversed by exogenous MT administration under the NaCl treatment, which dramatically increased ET_o_/RC while significantly decreasing ABS/RC, DI_o_/RC, and TR_o_/RC. The levels of ABS/RC, DI_o_/RC, and TR_o_/RC considerably rose in both the NaCl and NM treatments when p-CPA was administered; however, ET_o_/RC dramatically decreased.

ABS/CS_m_, TR_o_/CS_m_, and ET_o_/CS_m_ in tomato seedling leaves treated with NaCl showed substantial decreases of 20.0%, 15.8%, and 35.5%, respectively, when reaction centers were closed (t = *F*_m_) (Figure 7). In contrast, DI_o_/CS_m_ showed a notable rise of 13.2%. The NM treatment considerably raised ABS/CS^m,^ TR_o_/CS_m_, and ET_o_/CS_m_ while decreasing DI_o_/CS_m_ as compared to the NaCl treatment. Under NaCl stress, however, the NP treatment dramatically increased DI_o_/CS_m_ while decreasing ABS/CS_m_, TR_o_/CS_m_, and ET_o_/CS_m_. The use of p-CPA weakens the effect of MT on these parameters.

**Figure 8:**
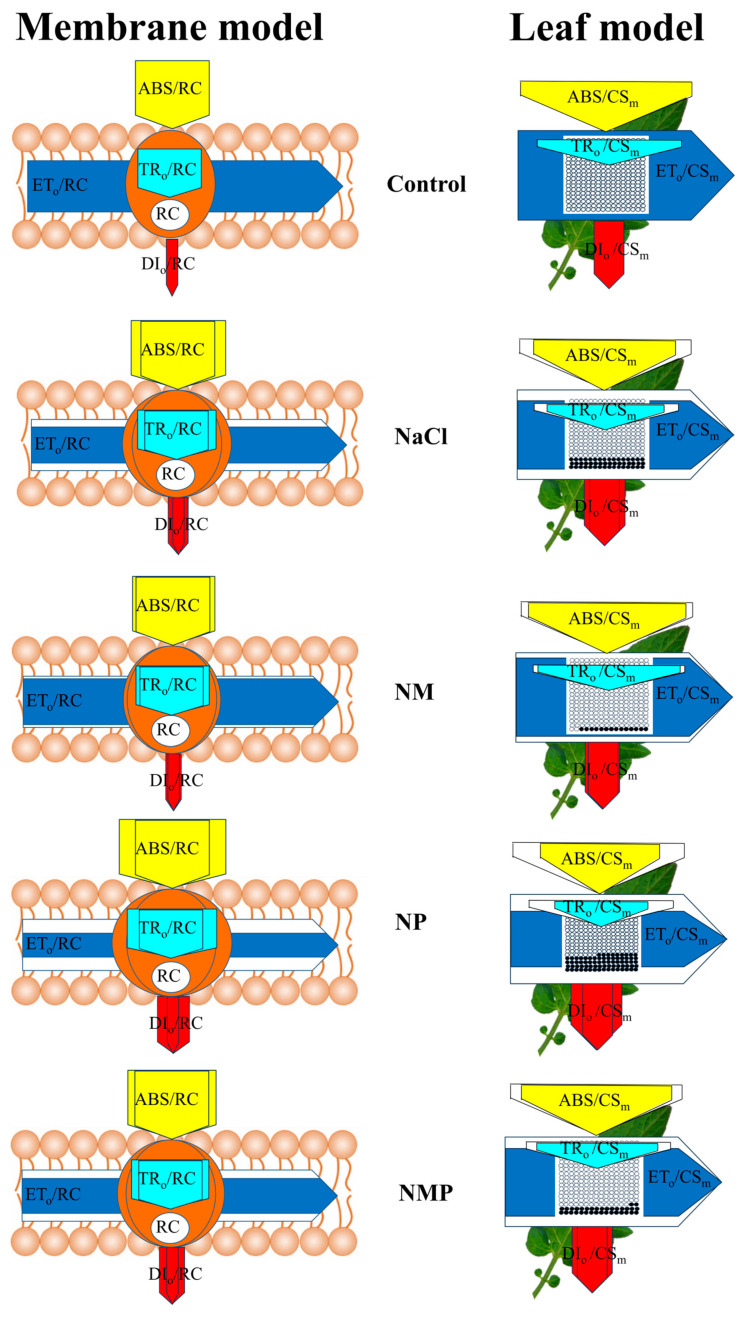
Energy pipeline models illustrating energy fluxes per PSII reaction center (RC) (left) and per excited cross-sectional area (CSm) (right) in leaves of tomato seedlings under salt stress, as influenced by exogenous melatonin (MT) application. Control: No NaCl, p-CPA, or MT added. NaCl: 100 mmol·L^−1^ NaCl applied alone. NM: 100 mmol·L^−1^ NaCl + 100 μmol·L^−1^ MT applied. NP: 100 mmol·L^−1^ NaCl + 100 μmol·L^−1^ p-CPA (MT synthesis inhibitor) applied. NMP: 100 mmol·L^−1^ NaCl + 100 μmol·L^−1^ MT + 100 μmol·L^−1^ p-CPA applied.

Figure 8 illustrates the particular activity parameter patterns of PSII across thylakoid membranes (left) and leaf tissues (right) for the Control, NaCl, NM, NP, and NMP treatments in order to further assess the effect of MT on the photosynthetic processes in tomato seedling leaves.

### 3.6. Effects of Exogenous MT on the PSI of Tomato Seedlings Under Salt Stress

**Figure 9:**
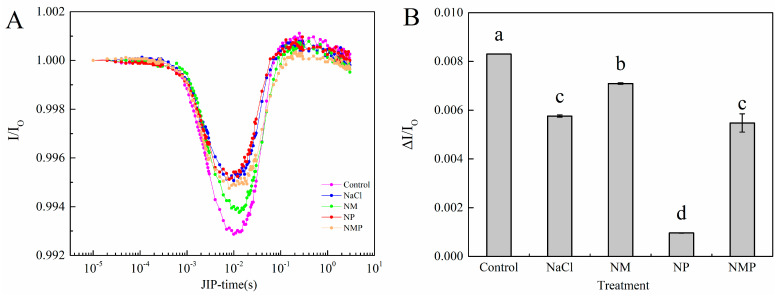
Curves of light-induced modulated 820 nm reflection kinetics (I/I_o_) (**A**), and ΔI/I_o_ (**B**) in leaves of salt-stressed tomato seedlings, as affected by exogenous melatonin (MT) application. Treatment differences are significant when indicated by different lowercase letters (*p* < 0.05). Control: No NaCl, p-CPA, or MT added. NaCl: 100 mmol·L^−1^ NaCl applied alone. NM: 100 mmol·L^−1^ NaCl + 100 μmol·L^−1^ MT applied. NP: 100 mmol·L^−1^ NaCl + 100 μmol·L^−1^ p-CPA (MT synthesis inhibitor) applied. NMP: 100 mmol·L^−1^ NaCl + 100 μmol·L^−1^ MT + 100 μmol·L^−1^ p-CPA applied.

An effective method for evaluating PSI complex activity under stress is the 820 nm light absorption curve. NaCl stress significantly decreased the maximum absorption of 820 nm in the tomato seedling leaves in comparison to the control, as seen in Figure 9A. On the other hand, the decrease in maximal PSI 820 nm absorption caused by NaCl was mitigated by the administration of exogenous MT. However, treatment with p-CPA further exacerbated the reduction in maximum 820 nm absorption under both the NaCl and NM treatments. In particular, ΔI/I_o_ was considerably decreased by 30.6% in comparison to the control when treated with NaCl. The administration of exogenous MT resulted in a 23.1% rise in ΔI/I_o_ relative to NaCl treatment, whereas p-CPA application caused a significant decrease of 83.3%. In comparison to the NM treatment, the NMP treatment significantly reduced ΔI/I_o_.

## 4. Discussion

Plant growth and development are severely restricted by salt stress, which also negatively impacts morphological structures and physiological-biochemical processes. In extreme or protracted cases, salt stress may result in plant death. An important metric for assessing how plants react to salt stress is biomass accumulation. NaCl stress considerably reduced tomato seedling development and biomass accumulation, according to this research (Figure 1). Nonetheless, the administration of exogenous melatonin (MT) significantly alleviated these inhibitory effects. These results align with earlier research that showed MT’s protective function in reducing growth suppression brought on by salt [43,44]. Additionally, the NMP treatment group showed that treatment with p-chlorophenylalanine (p-CPA), an inhibitor of melatonin production, reduced the protective benefits of exogenous MT and worsened the growth inhibition brought on by salt stress. We measured the expression of important genes involved in MT biosynthesis and endogenous MT levels in order to clarify the mechanism behind MT-mediated salt tolerance. Melatonin biosynthesis in plants is a complex process that involves numerous enzymatic stages. Genes such as *TrpDC*, *T5H*, *SNAcT*, and *AcSNMT* are essential for the regulation of this process [45,46]. These genes modulate endogenous MT levels in response to environmental stressors, as previous research has shown [47,48]. For instance, Wang et al. [49] found that elevated temperatures upregulated *SlSNAT*, which in turn improved tomato MT biosynthesis to provide heat resistance. In a similar vein, Jiao et al. [50] demonstrated that grapevines under salt stress had higher expressions of *VvTDC* and *VvSNAT*, which encouraged MT accumulation to prevent salt-induced damage. Conversely, we found that tomato seedlings under extended salt stress had lower endogenous MT levels, which may be because both MT synthesis and stability are inhibited. The observed downregulation of genes involved in MT biosynthesis during salt stress provided evidence in favor of this. The presence of p-CPA lessened this impact, whereas exogenous MT administration reversed this tendency by upregulating the expression of these genes, increasing endogenous MT levels. These findings imply that by modifying the expression of important MT biosynthesis genes, exogenous MT improves salt tolerance and restores endogenous MT levels (Figure 2).

Under stressful circumstances, photosynthesis—the main process by which plants accumulate dry matter—is essential to plant growth and development. Studies show that a decrease in photosynthetic ability is often strongly linked to a fall in plant development under stress [51,52]. In keeping with these results, we found that NaCl stress decreased the net photosynthetic rate (*P*_n_) in tomato seedlings, mostly because of non-stomatal causes, as shown by a reduction in stomatal conductance (*G*_s_) and an increase in intercellular CO_2_ (*C*_i_) (Figure 3). In contrast to p-CPA therapy, which intensified the inhibitory effects of salt stress and negated the mitigating effects of MT, exogenous MT administration successfully mitigated the detrimental effects of salt stress on *P*_n_, *C*_i_, *G*_s_, and transpiration rate (*T*_r_). This suggests that MT probably enhances photosynthesis under salt stress, mainly by resolving non-stomatal constraints such as reduced photochemical activity, chloroplast ultrastructure damage, and the degradation of chlorophyll pigment.

By using a kinetic model, the OJIP curve models a sequence of reduction processes in the electron transfer chain. Points that restrict the pace of electron transfer include the Q_B_ site in PSII and PQ, the re-oxidation of PQH_2_ at Cytb_6_f, and the flow of electrons between the two sites. As a result, the kinetic rise process is separated into three phases, which correspond to the OJ, JI, and IP portions of the OJIP curve and provide detailed information about PSII photochemical processes [53]. Consequently, to further examine the damage inflicted by NaCl stress on the photosynthetic apparatus in tomato seedling leaves and the mechanism through which exogenous MT enhances photosynthetic performance, this study utilized OJIP and 820 nm light absorption curves to analyze photochemical alterations in PSII prior to the activation of carbon assimilation. The shape of the OJIP curve deforms under salt stress, indicating that it is very sensitive to this condition [32]. This study’s findings corroborate this idea; as shown in Figure 4, the OJIP curve becomes flatter when subjected to salt stress because *F*_o_ (O phase) increases and *F*_m_ (P phase) decreases. Furthermore, J-phase fluorescence (V_J_) and I-phase fluorescence (V_I_) elevated after OJIP normalization (Figure 4B and Figure 6), signifying an augmented Q_A_ reduction level, whilst a drop in Sm indicates a diminished energy need for Q_A_ reduction. The reduction in energy necessary for Q_A_ redox and the rise in the extent of reduction suggest that the electron transfer to Q_B_ is obstructed, hence showing that the electron transfer process is impeded [54,55,56]. The K-band observed at 300 μs in the OJIP curve of NaCl-treated tomato leaves (Figure 5A) indicates impairment of the oxygen-evolving complex (OEC), consequently disturbing the balance between electron donation from the OEC to the oxidized PSII reaction center chlorophyll (P680^+^) and the re-oxidation of Q_A_- (the reduced PSII acceptor) [57]. The L-band seen at 150 μs in the OJIP curve of NaCl-treated tomato leaves signifies a disruption in the energy coupling (aggregation) of PSII units [58].

The whole photosynthetic system’s performance, including PSI and PSII, is reflected in PI_abs_ and PI_total_, which are performance indexes [59]. According to this research, salt stress decreased PI_abs_ and PI_total_ greatly (Figure 6). This suggests that salt stress interfered with PSII and PSI coordination, which severely affected the photosynthetic efficiency of the whole light system. The JIP parameters were computed in order to better identify the PSI and PSII damage sites [60]. While M_o_ and φD_o_ increased under salt stress, φP_o_, φE_o_, and ψ_o_ decreased (Figure 6). This suggests that PSII’s whole electron acceptor side in tomato seedling leaves, including the Q_A_, Q_B_, and PQ pool, was damaged, which hampered electron transport and exacerbated reaction center destruction. This validates earlier study results [61,62]. Additionally, salt stress decreased the likelihood of electron transfer from Q_B_ to PSI terminal electron acceptors (δR_o_), the reduction capacity of PSI terminal electron acceptors (φR_o_), and the maximal oxidation-reduction capacity of PSI (I/I_o_ and ΔI/I_o_) (Figure 6 and Figure 9). For this reason, we suggest that salt stress suppresses photosynthesis in tomato seedling leaves by breaking the complete electron transfer chain involved in photosynthetic processes from the PSII donor side to the PSI acceptor side. According to these findings, salt stress damage targets both the donor and acceptor sides of PSII and PSI. The effects of salt stress on the acceptor and donor sides of the photosynthetic system were successfully reversed by exogenous MT treatment, but exogenous p-CPA application worsened the damage caused by salt stress to the light system and lessened the protective benefits of MT. By reducing OEC damage, promoting electron transfer on the downstream side of Q_A_ under salt stress, increasing the electron transfer activity of PSII and PSI, and increasing the electron transfer flux of each reaction center (by increasing φP_o_, φE_o_, and ψ_o_, and decreasing M_o_ and φD_o_), these findings imply that MT plays a critical role in electron transfer from the PSII donor side to the PSI acceptor side.

To increase plant salt tolerance, PSII reaction center activity must be controlled, and the chloroplasts’ ability to collect and release energy must be improved [63]. In this study, DIo/CSm greatly rose whereas ABS/CS_m_, TR_o_/CS_m_, and ET_o_/CS_m_ dramatically reduced under salt stress (Figure 7 and Figure 8). The results indicate that the tomato leaves began appropriate defense mechanisms against salt stress, allowing PSII to promptly dissipate excess excitation energy. The unit active reaction centers’ energy absorption, conversion, and dissipation states, on the other hand, demonstrate that the inactivation of certain RCs suggests that the remaining active reaction centers must withstand significant light energy dissipation demands. Substantial increases in ABS/RC, DI_o_/RC, and TR_o_/RC and a substantial drop in ET_o_/RC were seen under salt stress (Figure 7 and Figure 8), indicating that the remaining active reaction centers’ light energy utilization and dissipation power were successfully improved. These outcomes align with earlier studies on tomatoes under salt stress [64]. By applying MT exogenously, the detrimental effects of salt stress on PSII RCs’ activity density, light energy absorption, and dissipation were successfully reduced. Nonetheless, the application of the MT synthesis inhibitor p-CPA intensified the damage to PSII reaction center activity caused by salt stress and negated the protective effects of MT. Yan [28] found that exogenous MT can promote the xanthophyll cycle and increase xanthophyll pool size under salt stress in rice to dissipate excess light energy. Additionally, the xanthophyll cycle plays a crucial protective role in limiting photodamage to the photosynthetic apparatus by reducing the transfer of captured excitation energy to PSII reaction centers [65]. Therefore, by controlling the xanthophyll cycle, MT in this research probably shields tomato seedlings from the negative consequences of excess excitation energy brought on by salt stress. These findings demonstrate that MT protects photosynthesis by lowering inactivated RCs in PSII, controlling energy capture, absorption, and thermal dissipation at PSII RCs, and enhancing the photochemical performance of active reaction centers. It also protects photosynthetic organs from salt stress. For this reason, MT is essential for improving tomato seedlings’ photoadaptation to salt stress.

## 5. Conclusions

To summarize, salt stress impairs the photosynthetic electron transport chain, which includes both photosystems II (PSII) and I (PSI), resulting in decreased photochemical activity, diminished reaction center functioning, and higher energy dissipation needs. These impacts hinder tomato seedling development and drastically reduce photosynthetic efficiency. Exogenous melatonin (MT) alleviates these adverse impacts by upregulating the expression of key MT biosynthesis genes, thereby increasing endogenous MT levels. Moreover, MT increases the activity of the OEC, restores PSI’s maximum redox capacity, and boosts the photochemical efficiency of PSII’s active reaction centers. These actions protect the donor and acceptor sides of PSII and PSI, facilitating smoother electron transport. Therefore, MT efficiently increases salt tolerance, boosts photosynthetic efficiency, and encourages tomato seedling development under salt stress.

## Data Availability

The original contributions presented in the study are included in the article; further inquiries can be directed to the corresponding authors.

## Supplementary Materials

The following supporting information can be downloaded at: https://www.mdpi.com/article/10.3390/plants14050824/s1, Table S1. Summary of Parameters and Formulae Derived from Chlorophyll a Fluorescence (OJIP) Transients. Table S2. Sequences of primers used for qPCR.
