# Supplementary material for: Melatonin Alleviates Photosynthetic Injury in Tomato Seedlings Subjected to Salt Stress via OJIP Chlorophyll Fluorescence Kinetics
Source: Plants (Basel). 2025 Mar 6;14(5):824. doi: 10.3390/plants14050824 (PMC11901964; doi:10.3390/plants14050824)
Supplement: Supplementary file 1 [file plants-14-00824-s001.zip › plants-3512016-supplementary.pdf]

plants

MDPI

**Table S1.** Summary of Parameters and Formulae Derived from Chlorophyll a Fluorescence (OJIP) Transients.

| Quick Chlorophyll Fluorescence Parameter | Description |
|---|---|
| **Parameters Calculated from JIP Test** | |
| $V_J=(F_J - F_o)/(F_m - F_o)$ | Relative variable fluorescence intensity at the J-step |
| $V_I=(F_I - F_o)/(F_m - F_m)$ | Relative variable fluorescence intensity at the I-step |
| $M_o=4(F_{300\mu s} - F_o)/(F_m - F_o)$ | Approximated initial slope of the fluorescence transient |
| $S_m=(Area)/(F_m - F_o)$ | Normalized total complementary area |
| **Quantum Yield or Energy Partitioning Ratio** | |
| $\varphi P_o=TR_o/ABS=[1-(F_o/F_m)]$ | Maximum quantum yield of primary photochemistry |
| $\varphi E_o=ET_o/ABS=[1-(F_o/F_m)]\psi_o$ | Quantum yield for electron transport (at t=$F_o$) |
| $\varphi D_o=1-\varphi P_o=(F_o/F_m)$ | Quantum yield at t=$F_O$ for energy dissipation |
| $\psi_o \equiv ET_o/TR_o=(1-V_J)$ | Probability that a trapped exciton moves an electron into the electron transport chain beyond $Q_A^-$(at t=$F_o$) |
| **Specific Activity Parameters (for $Q_A$ in a reducible state, per PSII reaction center)** | |
| $ABS/RC=M_o(1/V_J)(1/\varphi P_o)$ | Absorption flux per RC |
| $TR_o/RC=M_o(1/V_J)$ | Trapped energy flux per RC (at t=$F_o$) |
| $DI_o/RC=ABS/RC-TR_o/RC$ | Dissipated energy flux per RC (at t=$F_o$) |
| $ET_o/RC=M_o(1/V_J)(1/V_J)$ | Electron transport flux per RC (at t=$F_o$) |
| **Specific Activity Parameters (for illuminated material per unit area)** | |
| $ABS/CS_m \approx F_m$ | Absorption flux per CS(at t=$F$m) |
| $TR_o/CS_m=\varphi P_o(ABS/CS_m)$ | Trapped energy flux per CS (at t=$F_m$) |
| $DI_o/CS_m=(ABS/CS_m)-(TR_o/CS_m)$ | Dissipated energy flux per CS (at t=$F_m$) |
| $ET_o/CS_m=\varphi E_o(ABS/CS_m)$ | Electron transport flux per CS (at t=$F_m$) |
| **PSI Flux Ratio** | |
| $\delta R_o=RE_o/ET_o=(1-V_I)/(1-V_J)$ | Efficiency with which an electron from the intersystem electron carriers is transferred to reduce end electron acceptors at the PSI acceptor side |
| $\varphi R_o=RE/ABS=TR_o/ABS(1-V_I)$ | Quantum yield for reduction of end electron acceptors at the PSI acceptor side |
| $\Delta I/I_o=(I_{max}-I_{min})/I_o$ | Maximal redox capacity of PSI |
| **Performance Index** | |
| $PI_{abs}=RC/ABS[\varphi P_o/(1-\varphi P_o)][\psi_o/(1-\psi_o)]$ | Performance index on absorption basis |
| $PI_{total}=RC/ABS[\varphi P_o/(1-\varphi P_o)][\psi_o/(1-\psi_o)][\delta R_o/(1-\delta R_o)]$ | Performance index (potential) for energy conservation from exciton to the reduction of PSl end acceptors |

**Table S2.** Sequences of primers used for qPCR.

| Gene Name | ID | Primer | Sequence(5′ to 3′) |
|---|---|---|---|
| *Actin* | NM_001323002.1 | Forward | TGACTACGAGCAGGAACTTGAAACC |
| | | Reverse | AACGGAACCTCTCAGCACCAATG |
| *TrpDC* | Solyc07g054860.1.1 | Forward | GGTTTAACTGGCTGGCTTCG |
| | | Reverse | CATAGGATCGCTTCACTGGTTGTAC |
| *T5H* | Solyc09g014900.2.1 | Forward | CTCTTGACTACAATCCCGAAAG |
| | | Reverse | ACAAACGAGCCAACGATAAC |
| *SNAcT* | Solyc10g074910.1.1 | Forward | CGAGAAACTGATACGAACCCTT |
| | | Reverse | AGAACATTCCCTTAATTCCCTCTGG |
| *AcSNMT* | Solyc01g111900.2.1 | Forward | ATAGAGGCAATTCCAAAGACACAG |
| | | Reverse | GAGCAAGTCAAGGATAAGCCAGA |

Note: *Actin* (Internal control gene); *TrpDC* ( Tryptophan decarboxylase gene), *T5H* (Tryptamine 5-hydroxylase gene), *SNAcT* (Serotonin N-Acetyltransferase gene), *AcSNMT* (N-acetyl-serotonin methyltransferase gene).
